# The Challenge of Ecophysiological Biodiversity for Biotechnological Applications of Marine Microalgae

**DOI:** 10.3390/md12031641

**Published:** 2014-03-24

**Authors:** Lucia Barra, Raghu Chandrasekaran, Federico Corato, Christophe Brunet

**Affiliations:** Stazione Zoologica Anton Dohrn, Villa Comunale, Naples 80121, Italy; E-Mails: luciana.barra@szn.it (L.B.); raghu.chandrasekaran@szn.it (R.C.); federico.corato@szn.it (F.C.)

**Keywords:** biotechnology, biomass, diatoms, phytoplankton, ecophysiology, biodiversity, functional diversity, cultures, light

## Abstract

In this review, we aim to explore the potential of microalgal biodiversity and ecology for biotechnological use. A deeper exploration of the biodiversity richness and ecophysiological properties of microalgae is crucial for enhancing their use for applicative purposes. After describing the actual biotechnological use of microalgae, we consider the multiple faces of taxonomical, morphological, functional and ecophysiological biodiversity of these organisms, and investigate how these properties could better serve the biotechnological field. Lastly, we propose new approaches to enhancing microalgal growth, photosynthesis, and synthesis of valuable products used in biotechnological fields, mainly focusing on culture conditions, especially light manipulations and genetic modifications.

## 1. Introduction

It is now well known that marine biotechnology (the so-called “blue biotechnology”) can make a large contribution to the key societal challenges, in relation to the huge biological diversity populating marine ecosystems. As an alternative to higher plants, microbial organisms such as microalgae are relevant resources for applied societal purposes, due to their rich biodiversity, growth rate, and multiple application potentials.

Microalgae, either prokaryotes or eukaryotes, are oxygenic autotrophs that populate all aquatic ecosystems ranging from freshwater and brackish waters to oligotrophic marine waters. The microalgal world represents rich biodiversity, characterized by different biological, ecological and functional traits. The species number ranges from 30,000 described species to one million, and some estimates report more than 200,000 species only for the *Bacillariophyceae* [1,2]. This group is the most recent and diversified group [3], and until now, more than 8000‒10,000 diatom species have been described [4], while only a few species have been employed for biotechnological applications [5].

For Chlorophyceae, a group which comprises of almost 2000 species, only 7–8 species are biotechnologically active, followed by Cyanophyceae, where the number is even smaller [6,7]. Use of microalgae for biotechnological applications has been increasing in recent years, mainly for bioremediation, nutraceutical and pharmaceutical purposes, as well as for bioenergy production [8].

We can distinguish three kinds of algal applications (Table 1). The first application corresponds to the property of microalgae in interacting with the environment in which they grow, such as the use of biomass for O_2_ production, reduction of CO_2_ [9], bioremediation and bioremoval of some organic and inorganic compounds [10]. Microalgal species or strains are selected for their efficient growth or for their ability to harvest specific toxic compounds from the environment. The second application corresponds to the production of primary metabolites, as for instance carotenoids [11,12], proteins [13], and lipids such as polyunsaturated fatty acids, PUFA [14]. Some of these molecules have antioxidant activities [15,16,17]. For this purpose, microalgae with high physiological plasticity such as diatoms should be mainly used, since they are able to efficiently adapt to environmental variations [18]. The third application is related to secondary metabolites which are generally produced in low quantities, mainly for pharmaceutical applications [19].

Up to now, biotechnology uses around 20 species of microalgae from marine, estuarine and freshwater (lakes, ponds) ecosystems [6], representing a miniscule percentage of the myriad of known species. The open challenges for achieving the “green revolution” with “blue biotechnology” concern: (i) the cultivation of new species; (ii) identification of new molecules that can contribute to the development of industrial applications from marine ecosystems, and (iii) upscaling microalgal biomass production, while keeping production costs as low as possible. To overcome the aforementioned challenges, an extensive exploration of microalgal biodiversity (taxonomical and functional), chiefly by focusing on morphological, ecological and physiological traits, is mandatory. These traits, resulting from the adaptive evolution of a species to its ecological niche, determine its growth capacity in the marine environment. Many experimental results have shown that algal growth, physiology and biochemistry are strongly controlled by environmental variables, such as hydrology (temperature, salinity [20,21]), chemistry (pH, macronutrients and micronutrients [22]) and light [23]. Improving culture conditions will allow cells to be more efficient in terms of growth, photosynthesis and primary metabolite synthesis, since growth is the result of the energy dynamics balance between production and loss [24,25].

In this review we mainly focus on pelagic photosynthetic micro-organisms. The non-planktonic microalgae, while ecologically relevant, have different growth properties that we are not going to discuss in this paper (see the review by Lebeau and Robert [26]). This review aims to: (i) draw a general picture on the actual use of microalgae in the biotechnological field; (ii) disseminate the knowledge on biodiversity and richness of the microalgal world, and (iii) propose ways to improve microalgal production in the context of biotechnological applications. For the latter, we focus especially on the improvement of culturing conditions, in addition to the development of genetic engineering technologies [8].

## 2. Biotechnological Applications of Microalgae and Cultivation

### 2.1. Biotechnological Applications of Microalgae

Commercial culturing of algae has a history of 440 years, starting from the cultivation of *Porphyra* in the 1640s [7]. According to Hallmann [27], about 10^7^ tons of algae are harvested each year by algal biotechnology industries for different purposes, and almost 60 commercial companies are selling algae or algal products worldwide. In this section, we will go through their use as bio-markers/remediation, bio-fuel sources, and primary metabolite producers. Microalgae have gained the attention of the scientific world, as is shown in many studies, for their biochemical and physiological capacity to respond to organic pollutants (POPs) [28,29,30,31], polycyclic aromatic hydrocarbons (PHAs) [32,33,34], polychlorinated biphenyls (PCBs) [35,36] and pesticides [37,38,39,40,41]. These studies have demonstrated that macroalgae and microalgae are important tools in monitoring and controlling the presence of heavy metals in ecosystems, due to several biochemical strategies employed to reduce toxicity of non-essential trace metals [42,43,44,45,46,47,48]. Among the species resistant to heavy metal exposure, we can cite *Chlamydomonas reinhardtii* [49] and some macroalgae, such as *Fucus serratus*, alongside the aquatic plant *Lemna minor* [50].

Most organic chemicals can be naturally degraded within the aquatic environment as a result of complex processes mediated both by auto- and heterotrophic organisms [51]. However, when the wild-type xenobiotic detoxification systems, mainly based on metallothionein proteins [52,53], are not sufficient to cope with pollutant biodegradation, a possible alternative route is the creation of consortia made by microalgae and/or cyanobacteria [47,54]. A complete review of the mechanisms and solutions for pollutant management is provided by Torres and collaborators [28].

Many reports have described microalgae as a potential oxygen producer and CO_2_ depository [55,56], and as a green energy source for bio-fuels and biogases [57,58,59,60,61,62], providing feedstock for renewable fuels such as biodiesel, methane, hydrogen and ethanol. Moreover, microalgae grow faster and reach a higher productivity compared to conventional agricultural plants [59], and are able to grow almost anywhere, requiring only sunlight and nutrients [63,64,65]. This is discussed in greater detail in the fourth section of this review.

The wide amount of bioactive primary metabolite production is one of the key microalgal features to be exploited in many applicative fields, such as nutraceutics [66], animal feeding products and cosmetics [67]. Microalgal species, such as *Spirulina* for diet, *Dunaliella salina* and *Haematococcus pluvialis* for carotenoid production, and several other species for aquaculture, are used in mass culturing [68,69,70].

The high protein content, amino acid pools, carbohydrate digestibility, pigments, ω3 and ω6 family lipids and the presence of nearly all essential vitamins (e.g., A, B1, B2, B6, B12, C, E, nicotinate, biotin, folic acid and pantothenic acid) make microalgae an unconventional source for improving the food nutritional state and hence the health of humans and animals [13,66,71,72]. Furthermore, microalgae are considered to be of great interest in the biotechnological field because they are a precious source of lipophilic pigments such as chlorophyll (0.5% to 1% of dry weight), carotenoids (0.1% to 0.2% of dry weight on average and up to 14% of dry weight for β-carotene in some species, including *Dunaliella* sp.), xanthophylls (lutein, zeaxanthin and astaxanthin) and hydrophilic pigments, such as phycobiliproteins.

Recently, commercial forms of microalgal products are incorporated into pasta, snacks, gum, and beverages [73,74], as sources of natural food colorants, or as nutritional supplements [69,72,75]. The market is actually dominated by some species of chlorophytes and cyanophytes, such as *Arthrospira* spp., *Chlorella* spp., *Dunaliella salina*, *Heamatococcus pluvialis* and *Aphanizomenon flos-aquae*. More than 70 companies sell *Chlorella* as a source of β-1,3-glucan, which has properties of an immunosystem stimulator, as a free radical scavenger, and as a reducer of bad blood lipids [76]. *D. salina* is exploited for its β-carotene content that can reach 14% of its dry weight [77]. The chlorophyte *Muriellopsis* sp. is being exploited for the production of the xanthophyll lutein, due to its high content produced under peculiar culture conditions [78], while *Heamatococcus pluvialis* is used for its massive accumulation of astaxanthin, highly synthetized under stressful conditions [79,80]. Due to its high protein and amino acid content, *Arthrospira* is extensively used for human nutrition production in China and India, under the name of *Spirulina pacifica* [81,82]. According to several studies, the cyanophyte *A. flos-aquae* can also benefit health [83,84]. However, the diversity of usable species for aquaculture remains higher than the species that are used for human diets [85,86], since species such as *Phaeodactylum*, *Chaetoceros*, *Skeletonema* and *Thalassiosira*, along with *Chlorella*, *Tetraselmis*, *Isochrysis*, *Pavlova* and *Nannochloropsis*, are easily ingestible and digestible by cultivable organisms, and are used in aquaculture [87,88]. In the field of cosmetics, microalgal extracts are used for face, skin and hair care products, as well as in sunscreen products. Genera such as *Arthrospira* and *Chlorella* are employed in the skin care market [89]. For more information on companies selling microalgae and methods for their cultivation, see the review by Spolaore and collaborators [67].

Last, but not least, primary metabolites from microalgae are also used in diagnostics and application fields. In particular, phycobiliproteins, coloured proteins commonly present in cyanophytes and cryptophytes, are extensively commercialised in clinical and immunological analysis as colorants, fluorescent labelling molecules, and as pharmaceutical agents [90]. Until now, in the research and diagnostics development field, phycobiliproteins have been the object of more than 200 patents [90].

### 2.2. Algal Cultivation Techniques

The cultivation of microalgae can be performed indoor or outdoor, using enclosed or open systems [69]. These two systems strongly differ in shape, aeration, illumination systems, building material and volume capacity. In the past, natural waters (lakes, lagoons, *etc.*) or artificial ponds were used to grow microalgae. These outdoor open systems that use just natural light for illumination, are inexpensive to install and maintain and present uncertainties in the success of production operation. One of the main concerns is the uncontrolled environmental parameters, such as light, temperature, and air humidity that can induce large variability in cell physiology, altering the growth efficiency (see following sections). Excessive light or ultraviolet radiation in outdoor open systems might induce photoinhibition or can alter the healthy state of cells. Outdoor culturing for biomass production, with the exception of tropical areas, is strongly season dependent [91,92,93,94,95,96,97]. The transition periods of the year (e.g., end of spring and autumn) also show rapid climatological changes; a relevant temperature excursion between night and day might also affect physiological acclimation of microalgal biomass [98]. Other drawbacks, linked to the fact that cultures are open and not axenic, are correlated to the proliferation of bacteria, viruses, other microalgal species and predators that may decimate microalgal cultures [70]. Closed photo-bioreactors that can be set indoor or outdoor (either naturally or artificially illuminated), have been employed to axenically grow microalgae, such as cyanophytes [70,99,100]. For some diatoms, both outdoor and indoor cultivation systems have been established together with a generalised set of conditions [5]. The conditions and growth parameters can be set more accurately in indoor culture batches than in outdoor ones. Indoor culturing techniques prevent many environmental and biological causes of death or reduction in growth rate, and are more efficient for maintaining mass cultivation at high production rate. Section 4 of this review focuses on the possible ways to improve growth quantum yield of microalgal cultivation in indoor systems.

## 3. Exploring the Richness of Microalgal Biodiversity

Biodiversity is characterized by taxonomical metrics with distinct morphological traits, as well as other variables related to functional diversity such as ecology, physiology, and biochemistry of the species [101]. The cell functional traits regulate the ecophysiological requirements of the species, thereby shaping cell performances under specific conditions or along environmental gradients, as in nature. In this section, we aim to present the richness of microalgal biodiversity, which has been underexploited thus far in the biotechnological field.

### 3.1. Taxonomical Diversity

Biodiversity is huge in the microalgal realm, with almost 64 classes (phyla) and more than 70,000 species that could represent the basin to draw for a deep investigation of the microalgal world within a blue biotechnology aim [1]. The most recent and successful taxon group from an evolutionary and biogeochemical point of view is the diatoms’ group [3,102] that has been estimated to have roughly 10^5^ species [103]. Many diatoms comprise cryptic or hidden species not easily recognizable with classical microscopic tools [104,105]. For this reason in recent years, taxonomy has been using molecular tools, in addition to morphological identification to better decipher the richness of the microalgal realm [106] and the evolutionary trends of microalgae (e.g., [107,108,109,110]). Molecular tools allow for the investigation of small cell-sized species and uncultivable ones [111], as well as cryptic species [104,105,112]. As mentioned previously, for some taxa of phytoplankton, the known biodiversity does not take into account the hidden and cryptic species present in the groups [113]. Indeed, with the use of ribosomal or organelle markers as LSU, SSU, ITS, 5.8 S, RbcL and COX1 associated with the ultrastructure of a diatom cell wall, the silica frustule helps resolve cryptic species belonging to the following genera: *Chaetoceros*, *Skeletonema* and *Pseudo*-*nitzschia* [102,104,114] thereby enlarging the possibility of choosing the most appropriate species to the *ad hoc* cultivation system. For more information on taxonomical diversity and evolution of phytoplankton, we suggest the recent review papers [109,110].

### 3.2. Functional Diversity: Morphology and Size

Morphological variations amongst species lead cells to be more or less cultivable; these variations mostly imply changes in the surface: volume ratio, cell floatability and sinking rate. A cell’s shape can vary from round to elongated or triangular, affecting interactions between cells and the surrounding environment and having consequences also on the assimilation of resources [115].

The main cellular morphology includes the presence of flagella (Table 2), such as in the dinophytes, cryptophytes and some haptophytes, in addition to the presence of spines, referred to as setae and found mostly in diatoms. The setae can have several functions, such as cell protection against predators, sedimentation by trapping air bubbles and generation of photosynthetic oxygen. Indeed, setae from some *Chaetoceros* species contain chloroplasts that are able to move up and down, probably in relation to the light they are exposed to [116].

Another morphological trait is represented by the possibility of forming a colony. Colony-forming species could be considered evolutionarily favoured due to the colony-formation implication in defence mechanism (Table 2), as well as in growth regulation and competition [117]. One example is represented by some species of *Phaeocystis* (haptophyte) that can be present in both colonies and single cells [118].

Chain-forming species (Table 2) are also widespread in an aquatic environment, and mostly belong to the diatom genus [119]. Chain formation confers resistance to predation [120,121], enhances nutrient fluxes [122] and can increase sexual reproduction if chains become entangled [102].

The morpho-functional traits listed above are strongly ecologically relevant, though they do not add any *gain* for biotechnological purposes in relation to the current cultivation techniques that use high nutrient content and shallowness of culture tanks.

Instead, cell size can be considered as a relevant functional trait (Table 2), which has many implications for the ecophysiological requirement of cells and on the ecology of phytoplankton [101,123]. Cells range from 0.3 μm to several millimetres [123]. The smallest photosynthetic organisms, belonging to picophytoplankton class (size ˂3 μm) are strongly adapted to grow and dominate in oligotrophic waters [124,125]. The tiny cell size confers important ecological and biological properties on picophytoplankton, such as the large surface area per unit of volume, minimal diffusion boundary layer (*i.e.*, higher nutrient consumption rates), low sinking rate, low package effect and efficient light utilization [25,126,127]. Indeed, significant relationships between photosynthetic and growth rates were obtained in different picophytoplankton species, suggesting a direct and functional link between absorbed light [25,98] and metabolic processes. This could be related to a high physiological plasticity and intraspecific variability, as has been revealed in picophytoplankton [24,25,128,129,130]. These features might be related to a potentially higher rate of speciation in these small organisms than in larger sized ones [128].

**Table 1:** Discrimination of the three groups of algal applications.

Application	Function	Requirements	Algae
Biomass production	Fluxes of matter and energy	Optimization of culture conditions for growth and photosynthesis maximization	Large-sized coastal species, species with a high constitutive growth and photosynthesis
Primary metabolites	Production of interesting molecules such as carotenoids, phycobiliproteins, proteins, lipids, polysaccharides and antioxidants	Optimization of culture conditions for maximizing interesting molecules production and high growth rates (photosynthetic biotechnology through light manipulation and metabolic engineering)	Physiologically plastic species, such as small diatoms and coastal species
Secondary metabolites	Production of toxin or drugs	Optimal or stressful conditions to produce this kind of molecules	Selected or genetically modified species

**Table 2:** Functional traits in microalgae: Ecological or physiological relevancies and interests in a biotechnological field. * DMS: dimethylsulfide; ** DMSP: dimethylsulphoniopropionate; DCM: Deep-Chlorophyll Maximum.

Functional trait or adaptive feature	Ecological or physiological relevance	Group of species	Interests and/or problems in biotechnology
Multicellular life forms (chains and colony; [117])	Influence of sinking rate, reduced predation	Diatoms, haptophytes	Little impact in shallow and oxygenated/mixed tanks
Flagellates [131]	Migration and motility	Dinophytes, haptophytes and cryptophytes	Little impact in shallow and oxygenated/mixed tanks
Small cell size [127]	Low nutrient requirements, high growth capacity, low sinking rate	Picoeukaryotes	High growth and production capacity and acclimation
Benthic species [26,132]	Growth on solid support (sediments, leaves), highly resistant species	Some diatoms, cyanophytes	Difficult to cultivate
Toxic species [133]	Defence mechanisms, highly competitive	Cyanophytes, diatoms, dinophytes	Discovery and selection of new molecules
Sexual reproduction [134]	Genetic recombination	Some diatoms	New strains selection with better fitness
Diazotrophy [135]	Atmospheric N_2_ fixation	Cyanophytes	Low growth capacity
Mixotrophy [136,137,138]	Growth under nutrients depletion and darkness	Some dinophytes, diatoms, chrysophytes and cryptophytes	Low growth capacity, interest for bioremediation
Presence of large vacuoles [102]	Internal storage of nutrient	Diatoms	Long-term maintenance, decrease of dilution frequency
Low light adapted [139]	Growth under low light, photoinhibited under high light	Deep chlorophyll maximum (DCM) species	High growth rate under low light, high capacity of photoprotection
Variable light adapted [24]	Growth under low and high light, physiological plasticity	Coastal species (some diatoms and haptophytes)	High capacity of xanthophyll and antioxidant production, high growth rate
Low iron requirement species [140]	Growth in pelagic/oceanic ecosystems, photobiological and physiological adaptation	Some pennate diatoms	Little effect, Fe is provided in high quantity
Oceanic Temperature zones [141,142]	Low temperature growth	Polar species (diatoms, haptophytes)	Cost for low temperature maintenance of the cultures Temperature defence mechanisms, peculiar molecules for allowing photosynthesis and production at low temperature
Calcareous microalgae [143]	Species producing calcified scales around the cell	Coccolithophorids	Calcite production
DMSP, DMS producer species [15]	Antioxidant production (* DMS and ** DMSP) under environmental stresses	Prymnesiophytes, Diatoms, Dinophytes	Highly effective antioxidant systems, well-growing species
Halophilic species [144]	High salinity level, osmotic stress regulation	Chlorophytes and cyanophytes	Low growth capacity, costly culture management Molecules of interest

Although there is not much knowledge of picophytoplankton despite its high biodiversity, in addition to the features discussed earlier, it is implied that exploring picophytoplankton for biotechnological purposes is challenging. The low consumption together with the high growth rate and low loss of energy between light harvesting and division processes might outweigh the scant biomass content per cell.

### 3.3. Functional Diversity: Uptake of Nutritional Resources

The diversity of culture media available for growth of marine and coastal microalgae (Table 3) reflects the physiological diversity of microalgal groups. The major natural seawater-enriched media are: the f/2 medium, widely used for coastal and diatom species [145]; the K medium for oceanic species; the Pro99 medium for cyanophytes and picoeukaryotes; and the MNK medium mainly used for coccolithophorids [146].

**Table 3:** The most commonly used culture mediums for growth of marine microalgae (see also [147]).

Name	Microalgae	Specificities
f/2 medium [148,149]	Coastal microalgae, diatoms	Enriched medium
K medium [146,150]	Oceanic microalgae	Trace metals
Pro99 [151]	*Prochlorococcus* spp. and some picoeukaryotes	High ammonia concentrations, No vitamin requirement
MNK medium [152]	Oceanic coccolithophores	Enriched medium

The macronutrients required by microalgae to perform photosynthesis and growth are nitrogen (nitrate, NO_3_, nitrite, NO_2_ or ammonium, NH_4_), phosphorus (phosphate, PO_4_) and silicate (SiO_2_), the latter being required only for diatoms and silicoflagellates. As reported in many studies [101,153,154,155], the variability in uptake and the efficiency in using nutrients are high among phytoplankton groups, depending on evolutionary and ecological functional traits [156,157,158,159,160,161]. The most striking group corresponds to the diatoms, with the presence of a large central vacuole that can store nutrients and carbohydrates [102], which allows cells to maintain their growth during nutrient-depleted periods. This feature provides this group a competitive advantage over other groups.

Nitrogen can be provided as nitrate, nitrite or ammonium, which is a key variable for microalgal growth. It has been observed that different sources of nitrogen are differently metabolized, influencing the growth capacity and protein content [162], as observed in two *Chaetoceros* species [163]. Evidence from Meseck and collaborators [22] show that ammonium uptake efficiency in *Tetraselmis chui* PLY 429 significantly increased when carbon dioxide was added to the culture, while it decreased with a nitrate source. Experimental results obtained with the diatom *Skeletonema marinoi* grown in f/2 medium under different light conditions, revealed that after three or four days, ammonium concentration strongly decreased, while nitrite and nitrate concentrations remained high [164]. This result confirms that cells primarily use NH_4_, instead of NO_3_. Allen *et al.* [165] reported a decrease in growth rate of *Phaedactylum tricornutum* grown on nitrate as the only nitrogen source. In fact, the use of nitrate is more costly for cells than ammonium, because of the enzymes involved in its reduction into usable form [166,167]. However, high concentrations of ammonium have been shown to inhibit growth of some phytoplankton species, although the response is variable among the different groups [168]. Eppley *et al.* [169] did not show any differences on the half-saturation constants for nitrate and ammonium uptake among several coastal and oceanic species. Recently, the possible use of the intracellular NO_3_ by the benthic diatom *Amphora coffeaeformis* as a possible survival mechanism in darkness and anoxia has been shown [170]. The same mechanism has also been reported in the pelagic *Thalassiosira weissflogii* [171].

Macronutrient limitation affects growth capacity of microalgae, as well as their physiological state. Some of the physiological variations induced by a reduction in nutrients might be used for biotechnological applications. Nitrogen starvation in many microalgal species is commonly linked to an enhancement of triglyceride accumulation [61,172]. By contrast, lipid content tends to decrease with phosphorus limitation [173]; the latter reduces the formation of phospholipids and triggers the production of triglycerides and other neutral lipids [174]. N-limitation in addition to P-limitation induces an increase in non-photosynthetic carotenoids, as for instance astaxanthin in *Haematococcus pluvialis* [175], or β-carotene in *Dunaliella bardawil* upon nitrogen limitation [176]. Generally, nutrient limitation negatively affects photosynthesis and growth rates of microalgae [177,178,179,180,181,182]. P-limitation seems to repress the carbon-concentrating mechanism (CCM) activity and leads to lower photosynthesis [183], while N-limitation may have a stimulatory effect on the activity of the CCM, as shown in *Chlorella emersonii* [183].

Micronutrients, such as iron, manganese, zinc, cobalt, copper, molybdenum and the metalloid selenium, are also essential for microalgal growth. They are present in insoluble forms and can be limiting in oligotrophic waters [184,185]. To cope with this feature, offshore species have evolved lower iron requirements to survive in iron-poor oceanic waters compared to coastal species [186]. Iron is involved in controlling microalgal growth, as shown in the diatom *Cyclotella meneghiniana*, while its requirement is nitrogen source dependent [187,188]. Manganese is needed in large amounts for cells growing under low light conditions, as it is involved in photosynthesis [189]. Zinc is present in proteins involved in DNA transcription and in alkaline phosphatase; it is also used as a co-factor for carbonic anhydrase, a critical enzyme that transports and fixes CO_2_ [190]. Copper is also needed for photosynthesis, being required in cytochrome oxidase and plastocyanin, even though it can be substituted in some species by iron when cytochrome *c_6_* is present [191]. Nickel is a co-factor of urease, mainly required when urea is present as nitrogen source [192]. Other micronutrients, such as selenium, cadmium and mercury, can be added to the medium, albeit carefully because of their toxic effects. Moreover, three vitamins—vitamin B_12_ (cyanocobalamin), thiamine, and biotin—are also essential for microalgal growth [193].

Peculiar functional groups of microalgae concerning resource uptake are diazotrophs and mixotrophs (Table 2). Diazotrophs, primarily cyanophytes like the species *Trichodesmium*, fix atmospheric nitrogen and are able to grow without external sources of nitrogen. Beside a potential advantage of such strategy for biotechnological mass cultivation, the low metabolic rate efficiency of these species limits their applications in biotechnology [135]. Mixotrophs instead are able to perform both photosynthesis and heterotrophic grazing on particles or assimilate dissolved organic carbon (osmotrophy) [136,137,138]. Cells can therefore grow under inorganic nutrient depletion and this feature might favour the allocation of intracellular carbon into proteins [194]. This functional group can account for up to 20% of primary production, explaining plankton blooms under extreme conditions [195]. However, growth rate is generally low [196] and is strongly dependent on temperature, together with the efficiency of grazing activity. It has been recently shown that heterotrophic algae, such as *Scenedesmus* spp., can be used for fatty acid production [197]. Mixotroph species might be potentially used in the framework of the first kind of microalgae application, for pollutants bioremediation (Table 1).

### 3.4. Functional Diversity: Functional Traits versus Environmental Variables

Microalgae can also be characterized by their adaptive traits to optical, physical or hydrological variables. Since production is strictly related to the amount of energy supplied to the photosynthetic apparatus, light is the main variable driving photosynthetic capacity, and therefore growth. In aquatic ecosystems, light intensity is the most variable parameter, changing over different time and spatial scales and ranging from limiting to excessive. Light limitation influences nutrient uptake [198,199,200,201,202,203] and degree saturation of lipid [204,205]. Excessive light, which is damaging for cells, results in photoinhibition [206], in reduction of maximum quantum yield, and CO_2_ uptake [207,208,209]. Microalgae have developed protective mechanisms, to prevent or limit irreversible photoinhibition, in order to cope with the formation of reactive oxygen species (ROS) by synthesizing certain carotenoids or other molecules with antioxidant activities [66]. The first response to high light is the activation of photoprotective xanthophyll cycle [23], leading to the synthesis of zeaxanthin (in green algae) or diatoxanthin (in many chlorophyll *c*-containing algae). The role of these photoprotective xanthophylls, in countering peroxidative damage, has been reported in diatoms [210]. In *Haematococcus pluvialis* high light conditions increase the astaxanthin content three-fold as compared to low light [211,212].

Two functional groups, discriminated by light responses, have been proposed: high light and shade-acclimated species [139]. The high light group is characterized by low photosynthetic pigment content relative to the shade-acclimated species. More recently, Dimier *et al.* [24] discriminated three functional groups based on their photoregulation capacity: high light adapted, low light adapted and variable light adapted. The latter group, composed by coastal microalgae, is characterized by efficient and fast development of photoprotective processes such as xanthophyll cycle and non-photochemical quenching [24].

Recently, it has been shown that the growth capacity of the toxic *Ostreopsis ovata* (dinophyte) is strongly dependent on the effects of both temperature and light [21]. Temperature influences the growth of photoautotrophs through its control of enzymatic kinetics [213,214] and changes in cellular membrane composition [215]. Generally in microalgae, an increase in temperature leads to an enhancement in growth [216,217,218,219], as well as in carbon content [218,219,220,221,222]. Indeed, it has also been shown by Toseland and collaborators [142] that temperature strongly affects phytoplankton metabolism in the field as nutrients and light do. Microalgal responses to temperature also vary between groups (Table 2) [88,216,223,224]. The optimal temperature for growth capacity is usually below 10 °C for polar species [225,226], around 10–25 °C for temperate ones [227,228], close and beyond 25 °C for tropical species [88] and between 25 and 35 °C for desert-inhabiting microalgae [229].

Another relevant variable is salinity (Table 2). Osmoregulation is achieved by uptake of ions [230,231], the synthesis of osmotic active compounds or the expulsion of water [76,230,232,233,234,235]. Biochemical changes occur in cells exposed to high salinity, including an increase in ash content [236,237] and protein synthesis inhibition [238]. Estuarine species are tolerant to a wide range of salinities, though fewer species are able to grow in high salinity conditions. The success of these halophytic species is related to enhanced synthesis of osmotic stress regulation molecules such as the zeaxanthin in cyanophytes [239] or glycerol in the green alga *Dunaliella salina* [240]. The latter also accumulates β-carotene under high salinity stress [176,241,242] while *Haematococcus pluvialis* accumulates astaxanthin [175]. These species are already used in biotechnology to produce stress-response molecules, useful in cosmetics or nutrition [144].

In a culture batch, it is also important to monitor pH since it can vary in relation to photosynthesis. Moreover, pH variations can induce: (i) the differential availability of inorganic carbon sources (CO_2_/HCO_3_^−^/CO_3_^2−^) [243,244,245]; (ii) the co-precipitation of phosphate with calcium, magnesium and carbonate at high carbonate concentrations; (iii) the low solubility and availability of trace metals at high pH [246]; (iv) intracellular pH regulation; and (v) changes in the uptake of essential nutrients, such as nitrate and phosphate [22,247,248]. The diatom *Phaeodactylum tricornutum* presents high plasticity to pH variation being able to grow at pH > 10, making it one of the most common contaminants in poorly buffered cultures [249,250]. This property could be advantageous in outdoor mass cultivation.

## 4. Enhancing Production and Growth: “Photosynthetic Regulation Biotechnology” and Genetic Modifications

**Figure 1:**
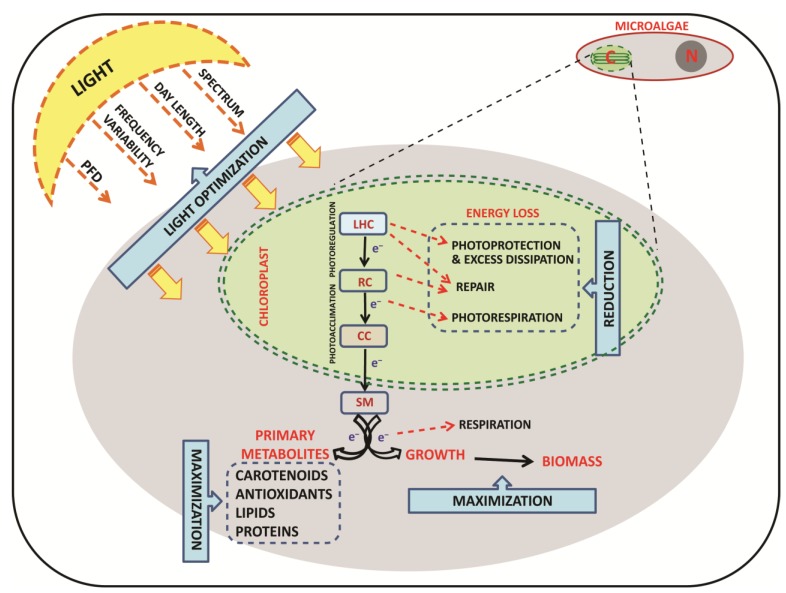
“Photosynthetic Regulation Biotechnology”: light manipulation to maximize photosynthesis and growth. The optimization of light in terms of photon flux density, spectral radiations, photoperiod, and frequency variability is investigated in order to reduce energy losses during the photosynthetic process and maximize biomass and primary metabolite production. PFD-Photon Flux Density; e^−^-electron; LHC-Light Harvesting Complex; RC-Reaction Center; CC-Calvin Cycle; SM-Storage Molecules; C-Chloroplast and N-Nucleus.

One of the main challenges for increasing biotechnological applications of microalgae is the enhancement of their growth and yield. The growth rate of a microalgal population is the result of the balance between actively dividing and death cells. This “performance index” mainly depends on both cells’ acclimation to the culture conditions and the energetic cost of internal processes regulation. The latter depends on how ecophysiological requirements match the environmental conditions and on physiological plasticity of the cultivated strains. Two of the most promising routes to progress, in enhancing biomass or interesting molecules production, are the “Photosynthetic Regulation Biotechnology” (Figure 1) and the genetic engineering of strains.

### 4.1. Light Control and the “Photosynthetic Regulation Biotechnology”

Light is the most relevant trigger for photosynthesis even though it is the most variable parameter at sea over different temporal and spatial scales (see previous section). Many recent studies, using physiological, biochemical or molecular approaches, have shown how light intensity, variability and spectral composition affect the physiological state and growth of cells [24,25,251,252,253]. By consequence, light manipulation might be a way for modifying microalgal productive performance [254,255], and therefore a relevant issue for biotechnological purposes (see the review by Murchie and collaborators [256]), in the field of “photosynthetic regulation biotechnology”.

Photosynthesis is a unique process that converts light energy into biochemical energy, through light and dark reactions. In response to light intensity variations, cells modulate two stages of the photosynthetic process by modifying the photosynthetic apparatus structure and pigments, as well as the enzymes involved in carbon fixation [257]. Recent studies have shown that these two steps do not respond in the same way to light changes, showing an uncoupling of their regulative processes that depend on the ecophysiological properties of cells, and this also affects the growth rate maintenance [25]. Photosynthetic properties and growth can be poorly coupled, as observed in a diatom and a prasinophyte subjected to fluctuating irradiance [258], reinforcing the observations on the variability of the relationship between photosynthetic organic production and cell growth [259]. This variability can be attributed to many factors, such as variations in respiration, changes in dissolved organic losses and metabolite storage, as for instance polysaccharides [258]. The discrepancy between growth and production is less severe in small cells with smaller intracellular energy storage [25,126,251].

The term “light” includes different key variables (Figure 1): instantaneous photon flux density (PFD), daily light dose intensity, photoperiod, light distribution, frequency of PFD variability and spectral characteristics [20,260]. The integrated daily light dose depends on the photoperiod and light distribution. The daily light dose experienced by cells under sinusoidal light distribution compared to quadratic shape is around 1.9 times less, when provided using the same 12:12 h light–dark photoperiod and maximal light value [164]. Light is generally provided following a quadratic distribution, *i.e.*, with an “on/off” switch system. Recent studies have shown that applying a sinusoidal light regime allows cells to activate a gradual and efficient photoregulation on the contrary of a quadratic distribution [24,251]. It has been observed that the slow increase of light at dawn allows cells to efficiently perform photoregulation and prepares the photosynthetic apparatus to cope with the high light midday peak [24,251]. By contrast, a constant excessive light induces considerable damage, increasing the biochemical costs associated with defence, protection and recovery, and decreasing the energy fuelled towards growth. Reciprocally, when constant light is limiting for optimal photosynthesis, cell performance will be reduced.

The photoperiod, *i.e.*, the succession of illuminated and dark periods during the day, influences microalgal growth and photosynthetic rate [20,261]. It is known that biomass synthesis, in terms of produced carbon, may be higher under continuous light than alternating light–dark phases [203,262]. However, cells do not appear healthy under continuous light [263]; the light–dark succession allows cells to recover and uncouple many biological processes, such as photosynthesis, from cell division [20,264]. Moreover, photosystem repairing and relaxing occur during the dark period; it is also known that during dark phytoplankton cells uptake and assimilate NH_4_ [265,266].

The very fast frequency of light variability (scale of milliseconds) positively influences photosynthetic efficiency, due to the re-oxidation of the electron transporters of the photosynthetic apparatus during dark phases [261]. Again, the frequency of the light variability, on an hour scale, is highly relevant. The shade acclimation state of cells is strengthened by the enhancement of the light fluctuations experienced, driving production of different carotenoids and antioxidants [251]. Light fluctuation dynamics also strongly affect the growth capacity of cells [251,258,259,267].

The spectral radiation of light does influence growth, photoacclimation state, and cell biochemistry [268,269]. Blue light affects many physiological processes in algae, such as photo-morphogenesis [270], chloroplast movement [271], cell division and photosynthetic acclimation in diatoms [252,253,270,272]. The spectral composition of light plays a key role in the ability of diatoms to finely balance light harvesting and photoprotective capacities [273]. Indeed, red radiation mixed to blue is necessary for activating the photoprotective pathway, such as the synthesis of xanthophyll pigments with an antioxidant role [274,275]. The spectral properties of microalgal absorption are the base for designing new models of photobioreactors and improving microalgal growth [276].

### 4.2. Genetic Transformations

Until now, biotechnological production from microalgae, such as food additives, cosmetics, animal feed additives, pigments, polysaccharides and fatty acids, is done in a non-transgenic way. However, genetic engineering is already being applied to microalgal research field, and selectable marker genes, promoters, reporter genes, transformation techniques, together with other genetic tools are available for various species. At present, about 20 species are accessible to genetic transformation [27], and large-scale sequencing projects are in progress for several microalgae species [277]. Sequences are available at the NCBI organelle database [278] and at the Organelle Genome Database [279].

The complete genome for about 30 cyanobacteria have already been sequenced; genome sequences of the cyanophyte *Synechococcus* [280], the green alga *Micromonas* [281] and *Ostreococcus* [128] are also available. For a more exhaustive list, please refer to the website from the Roscoff Culture Collection [282,283]. The following species’ genomes (nuclear, mitochondrial and plastidial): *Alexandrium tamarense, Amphidinium operculatum, Aureococcus anophagefferens*, *Chlorella vulgaris*, *Cyanophora paradoxa,* and *Dunaliella salina* are being sequenced, and 37 microalgae transcriptomic projects are currently taking place [8,284].

In the last few years, about 20 new microalgal species have been genetically modified with success: nine green microalgae, five diatoms, three cyanobacteria, two dinophytes, two red microalgae and one euglenoid have been successfully transformed; most of these were achieved by nuclear and stable transformation [27]. Stable transformation of four diatom species (*Phaeodactylum tricornutum, Navicula saprophila, Cylindrotecha fusiformis* and *Cyclotella cryptica*) has also been reported, and 62 promoters have already been tested for microalgae transformations [8]. The use of engineered nucleases to genetically reprogram diatoms, with the aim of producing bio-fuels, has been successfully demonstrated [285]. By targeting specific sequences within diatoms’ genomes, nucleases can be used to accurately insert, correct, or inactivate specific genes. With the whole genome sequencing of several diatom species, such as *Thalassiosira pseudonana* and *Phaeodactylum tricornutum*, a new era of post-genomics research has begun, with opportunities to improve fundamental understanding of the diatoms biology, and to build a molecular foundation for new industrial applications. Stable mutants of some species of microalgae have been obtained in the last years such as *Phaeodactylum tricornutum*, expressing a heterologous functional glucose transporter, *Dunaliella salina* (*zea1*) overproducing zeaxanthin, and cyanobacteria-synthetizing mosquito larvacides [7,27,80,286,287,288,289]. *Euglena gracilis* has been transformed at the chloroplast level [290], while *Thalassiosira weissflogii* has been only transiently transformed [287]. Some groups are more difficult to be transformed by electroporation or conjugation [291], as for instance diatoms with their silica wall, compared to species from other groups such as *Spirulina*, *Anabaena*, or *Synechocystis*. Recently *Synechocystis* sp PCC6803 was deeply analysed, through several growth-coupled knockouts, to identify its main metabolic properties in the aim of biotechnological applications [292].

One of the main goals in biotechnological genetic engineering is their use as “bioreactors” in producing vaccines [293,294,295], specific proteins [296], or bioenergetic molecules [297]. Some therapeutic proteins have been successfully produced using microalgae, mainly the Chlorophyta *Chlamydomonas reinhardtii*, for which suitable transgenic tools and genomic data are available (for all three genomes: nuclear, chloroplastic and mitochondrial). The chloroplast of *C. reinhardtii* has been used to produce a range of recombinant proteins, including reporters such as glucuronidase (GUS), luciferase (LUC), green fluorescent protein (GFP), industrial enzymes, vaccines and therapeutic enzymes [298]. To date [8], 18 biopharmaceutical proteins have been expressed in *C. reinhardtii* and one in *Chlorella ellipsoidea* [298,299]. Diatoms have not been employed for expression of any biopharmaceutical proteins, but Hempel and collaborators [300] have recently reported the first stable expression of a full-length human antibody and the respective antigen in *P. tricornutum*.

In the bio-fuel production field, the Heterokonta *Nannochloropsis* represents a new model with potential for further development into an integrated photons-to-fuel production platform [301]. Several isolates of *Nannochloropsis* spp. produce large quantities of triacylglycerols, related to over-representation of genes involved in lipid biosynthesis together with rapid growth capacity, and industrial-scale cultivation [301]. The genome of the high PUFA-content species *Nannochloropsis oceanica* has been recently sequenced by Pan and collaborators [302]. Sequence similarity-based investigation identified new elongase- and desaturase-encoding genes involved in the biosynthesis of PUFAs, which provide a genetic basis for its rich eicosapentaenoic acid (EPA) content, making this species suitable for the genetic engineering of a triacylglycerols pathway.

To conclude, we can assert that genetic transformation could be a valid tool to “revolutionize” blue biotechnology. However, cautious measures have to be taken for ecosystem bio-safety and to monitor transgenic microorganisms in nature.

## 5. Conclusions

The functional biodiversity of microalgae has to be explored thoroughly, and mass culturing conditions presently used have to be revisited in order to optimize the fitness of cultivated species and decrease production costs. Indoor culturing systems would be preferable to outdoor systems for the reasons already discussed on the controlled *vs.* uncontrolled environmental parameters. We also suggest use of local microalgal species and seawater from which the species have been isolated, *i.e*., cultivation next to aquatic ecosystems, in the frame of what we can call the “Km-0 mass cultivation” strategy. This strategy might reduce the cost of cultivation, allow for the use of freshly isolated species and strains, and potentially also provide high flexibility in species choice that share common ecophysiological requirements. This could also solve the potential loss of growth efficiency due to long-term cultivation maintenance.

Furthermore, we recommend the use of ammonium, as nitrogen source, to increase biomass production, since it is assimilated faster than nitrate, and this allows an increase in the production efficiency (see above). We suggest providing light with an intra-diel light–dark cycle, with a sinusoidal shape instead of quadratic distribution, for the reasons discussed in the previous sections. Turbulence, reproducing coastal ecosystems, can be applied to coastal species cultivation, through a light variation frequency program. We propose the use of illumination systems that allow regulation of the photon flux density and the spectral composition to better manage photosynthetic productivity. For many microalgal groups (e.g., diatoms or chlorophytes), green radiation may be excluded from the light spectrum since it is not harvested, in order to increase the growth yield and reduce production costs [276]. Since the effect of light variations on physiology and growth of autotrophs is relevant, we suggest that genetic engineering should mainly target the photophysiological response system (e.g., [252,253,303]), entering the “photosynthetic regulation biotechnology.” The photophysiological pathway transformation of microalgae could be useful but with a high probability of obtaining a productive strain with a slow growth.

Diatoms that include many coastal species might be the ideal model to cultivate with biotechnological aims, for many reasons, such as the huge biodiversity of this group and many biological peculiarities (see previous). Many diatom species grow on benthic substrates, at least during one step of their life cycle; therefore, their cultivation for biotechnological purposes needs a deeper communication between ecophysiological and process engineering researchers. Moreover, they are able to reproduce sexually [134], allowing this group frequent gene recombination processes and present high physiological flexibility [274]. Due to this relevant feature, we could easily manipulate their photophysiological responses and biochemical pathways to increase the production of targeted molecules by varying the culture conditions.

## References

[1] Guiry M.D. (2012). How many species of algae are there?. J. Phycol..

[2] Public Algaebase: Listing the World’s Algae. http://www.algaebase.org.

[3] Armbrust E.V. (2009). The life of diatoms in the world’s oceans. Nature.

[4] Tomas C.R. (1997). Identifying Marine Phytoplankton.

[5] Lebeau T., Robert J.M. (2003). Diatom cultivation and biotechnologically relevant products. Part II: Current and putative products. Appl. Microbiol. Biotechnol..

[6] Chu W.-L. (2012). Biotechnological applications of microalgae. IeJSME.

[7] Pulz O., Gross W. (2004). Valuable products from biotechnology of microalgae. Appl. Microbiol. Biotechnol..

[8] Cadoret J.-P., Garnier M., Saint-Jean B. (2012). Microalgae, Functional Genomics and Biotechnology. Adv. Bot. Res..

[9] Zeiler K.G., Heacox D.A., Toon S.T., Kadam K.L., Brown L.M. (1995). The use of microalgae for assimilation and utilization of carbon-dioxide from fossil fuel-fired power plant flue gas. Energy Convers. Manag..

[10] Wilde E.W., Benemann J.R. (1993). Bioremoval of heavy-metals by the use of microalgae. Biotechnol. Adv..

[11] Guedes A.C., Amaro H.M., Malcata F.X. (2011). Microalgae as sources of carotenoids. Mar. Drugs.

[12] Christaki E., Bonos E., Giannenas I., Florou-Paneri P. (2013). Functional properties of carotenoids originating from algae. J. Sci. Food Agric..

[13] Becker W., Richmond A. (2004). Microalgae in Human and Animal Nutrition. Handbook of Microalgal Culture.

[14] Sharma K.K., Schuhmann H., Schenk P.M. (2012). High lipid induction in microalgae for biodiesel production. Energies.

[15] Sunda W., Kieber D.J., Kiene R.P., Huntsman S. (2002). An antioxidant function for DMSP and DMS in marine algae. Nature.

[16] Stahl W., Sies H. (2003). Antioxidant activity of carotenoids. Mol. Aspects Med..

[17] Munir N., Sharif N., Naz S., Manzoor F. (2013). Algae: A potent antioxidant source. Sky J. Microbiol. Res..

[18] Dubinsky Z., Stambler N. (2009). Photoacclimation processes in phytoplankton: Mechanisms, consequences, and applications. Aquat. Microb. Ecol..

[19] Borowitzka M.A. (1995). Microalgae as sources of pharmaceuticals and other biologically-active compounds. J. Appl. Phycol..

[20] Grobbelaar J.U. (2010). Microalgal biomass production: Challenges and realities. Photosynth. Res..

[21] Scalco E., Brunet C., Marino F., Rossi R., Soprano V., Zingone A., Montresor M. (2012). Growth and toxicity responses of Mediterranean *Ostreopsis cf. ovata* to seasonal irradiance and temperature conditions. Harmful Algae.

[22] Meseck S.L., Smith B.C., Wikfors G.H., Alix J.H., Kapareiko D. (2007). Nutrient interactions between phytoplankton and bacterioplankton under different carbon dioxide regimes. J. Appl. Phycol..

[23] Brunet C., Johnsen G., Lavaud J., Roy S., Roy S., Johnsen G., Llewellyn C., Egeland E.S. (2011). Pigments and Photoacclimation Processes. Phytoplankton Pigments, Characterization, Chemotaxonomy and Applications in Oceanography.

[24] Dimier C., Saviello G., Tramontano F., Brunet C. (2009). Comparative ecophysiology of the xanthophyll cycle in six marine phytoplanktonic species. Protist.

[25] Giovagnetti V., Cataldo M.L., Conversano F., Brunet C. (2012). Growth and photophysiological responses of two picoplanktonic *Minutocellus* species, strains RCC967 and RCC703 (Bacillariophyceae). Eur. J. Phycol..

[26] Lebeau T., Robert J., Rao S. (2006). Biotechnology of immobilized micro algae: A Culture Technique for the Future?. Algal Cultures, Analogues of Blooms and Applications.

[27] Hallmann A. (2007). Algal transgenics and biotechnology. Transgenic Plant. J..

[28] Torres M.A., Barros M.P., Campos S.C.G., Pinto E., Rajamani S., Sayre R.T., Colepicolo P. (2008). Biochemical biomarkers in algae and marine pollution: A review. Ecotoxicol. Environ. Saf..

[29] Montone R.C., Taniguchi S., Weber R.R. (2001). Polychlorinated biphenyls in marine sediments of Admiralty Bay, King George Island, Antarctica. Mar. Pollut. Bull..

[30] Leitão M.A.d.S., Cardozo K.H.M., Pinto E., Colepicolo P. (2003). PCB-Induced oxidative stress in the unicellular marine dinoflagellate *Lingulodinium polyedrum*. Arch. Environ. Contam. Toxicol..

[31] Gerofke A., Kömp P., McLachlan M.S. (2005). Bioconcentration of persistent organic pollutants in four species of marine phytoplankton. Environ. Toxicol. Chem..

[32] Aksmann A., Tukaj Z. (2004). The effect of anthracene and phenanthrene on the growth, photosynthesis, and SOD activity of the green alga *Scenedesmus armatus* depends on the PAR Irradiance and CO_2_ level. Arch. Environ. Contam. Toxicol..

[33] Djomo J.E., Dauta A., Ferrier V., Narbonne J.F., Monkiedje A., Njine T., Garrigues P. (2004). Toxic effects of some major polyaromatic hydrocarbons found in crude oil and aquatic sediments on *Scenedesmus subspicatus*. Water Res..

[34] Lei A.-P., Hu Z.-L., Wong Y.-S., Tam N.F.-Y. (2007). Removal of fluoranthene and pyrene by different microalgal species. Bioresour. Technol..

[35] Doick K.J., Klingelmann E., Burauel P., Jones K.C., Semple K.T. (2005). Long-term fate of polychlorinated biphenyls and polycyclic aromatic hydrocarbons in an agricultural soil. Environ. Sci. Technol..

[36] Borja J., Taleon D.M., Auresenia J., Gallardo S. (2005). Polychlorinated biphenyls and their biodegradation. Process Biochem..

[37] Geoffroy L., Teisseire H., Couderchet M., Vernet G. (2002). Effect of oxyfluorfen and diuron alone and in mixture on antioxidative enzymes of *Scenedesmus obliquus*. Pestic. Biochem. Physiol..

[38] Nyström B., Becker-Van Slooten K., Bérard A., Grandjean D., Druart J.-C., Leboulanger C. (2002). Toxic effects of Irgarol 1051 on phytoplankton and macrophytes in Lake Geneva. Water Res..

[39] Geoffroy L., Dewez D., Vernet G., Popovic R. (2003). Oxyfluorfen toxic effect on *S. obliquus* evaluated by different photosynthetic and enzymatic biomarkers. Arch. Environ. Contam. Toxicol..

[40] Ma J., Chen J. (2005). How to accurately assay the algal toxicity of pesticides with low water solubility. Environ. Pollut..

[41] Cai X., Liu W., Jin M., Lin K. (2007). Relation of diclofop-methyl toxicity and degradation in algae cultures. Environ. Toxicol. Chem..

[42] Hall J.L. (2002). Cellular mechanisms for heavy metal detoxification and tolerance. J. Exp. Bot..

[43] Conti M.E., Cecchetti G. (2003). A biomonitoring study: Trace metals in algae and molluscs from Tyrrhenian coastal areas. Environ. Res..

[44] Pinto E., Sigaud-kutner T.C.S., Leitão M.A.S., Okamoto O.K., Morse D., Colepicolo P. (2003). Heavy metal induced oxidative stress in algae. J. Phycol..

[45] Nishikawa K., Yamakoshi Y., Uemura I., Tominaga N. (2003). Ultrastructural changes in *Chlamydomonas acidophila* (Chlorophyta) induced by heavy metals and polyphosphate metabolism. FEMS Microbiol. Ecol..

[46] Mallick N. (2004). Copper-induced oxidative stress in the chlorophycean microalga *Chlorella vulgaris*: Response of the antioxidant system. J. Plant Physiol..

[47] Perales-Vela H.V., Peña-Castro J.M., Cañizares-Villanueva R.O. (2006). Heavy metal detoxification in eukaryotic microalgae. Chemosphere.

[48] Tripathi B.N., Mehta S.K., Amar A., Gaur J.P. (2006). Oxidative stress in *Scenedesmus* sp. during short- and long-term exposure to Cu^2+^ and Zn^2+^. Chemosphere.

[49] Schroda M., Vallon O., Wollman F.A., Beck C.F. (1999). A chloroplast-targeted heat shock protein 70 (HSP70) contributes to the photoprotection and repair of photosystem II during and after photoinhibition. Plant Cell.

[50] Ireland H.E., Harding S.J., Bonwick G.A., Jones M., Smith C.J., Williams J.H.H. (2004). Evaluation of heat shock protein 70 as a biomarker of environmental stress in *Fucus serratus* and *Lemna minor*. Biomarkers.

[51] Singer A.C., Thompson I.P., Bailey M.J. (2004). The tritrophic trinity: A source of pollutant-degrading enzymes and its implications for phytoremediation. Curr. Opin. Microbiol..

[52] Lewis S., Donkin M.E., Depledge M.H. (2001). Hsp70 expression in *Enteromorpha intestinalis* (Chlorophyta) exposed to environmental stressors. Aquat. Toxicol..

[53] Spijkerman E., Barua D., Gerloff-Elias A., Kern J., Gaedke U., Heckathorn S. (2007). Stress responses and metal tolerance of *Chlamydomonas acidophila* in metal-enriched lake water and artificial medium. Extremophiles.

[54] Andrade L.R., Farina M., Amado Filho G.M. (2004). Effects of copper on *Enteromorpha flexuosa* (Chlorophyta)* in vitro*. Ecotoxicol. Environ. Saf..

[55] Chinnasamy S., Bhatnagar A., Claxton R., Das K.C. (2010). Biomass and bioenergy production potential of microalgae consortium in open and closed bioreactors using untreated carpet industry effluent as growth medium. Bioresour. Technol..

[56] Sayre R. (2010). Microalgae: The potential for carbon capture. BioScience.

[57] Sheehan J., Dunahay T., Benemann J., Roessler P. (1998). A Look Back at the US Department of Energy’s Aquatic Species Program.: Biodiesel from Algae.

[58] Tsukahara K., Sawayama S. (2005). Liquid fuel production using microalgae. J. Jpn. Pet. Inst..

[59] Chisti Y. (2007). Biodiesel from microalgae. Biotechnol. Adv..

[60] Li Y., Horsman M., Wu N., Lan C.Q., Dubois-Calero N. (2008). Biofuels from microalgae. Biotechnol. Prog..

[61] Li Y., Horsman M., Wang B., Wu N., Lan C. (2008). Effects of nitrogen sources on cell growth and lipid accumulation of green alga *Neochloris oleoabundans*. Appl. Microbiol. Biotechnol..

[62] Mata T.M., Martins A.A., Caetano N.S. (2010). Microalgae for biodiesel production and other applications: A review. Renew. Sustain. Energ. Rev..

[63] Renaud S.M., Thinh L.V., Parry D.L. (1999). The gross chemical composition and fatty acid composition of 18 species of tropical Australian microalgae for possible use in mariculture. Aquaculture.

[64] Pratoomyot J., Srivilas P., Noiraksar T. (2005). Fatty acids composition of 10 microalgal species. Songklanakarin J. Sci. Technol..

[65] Aslan S., Kapdan I.K. (2006). Batch kinetics of nitrogen and phosphorus removal from synthetic wastewater by algae. Ecol. Eng..

[66] Jin E., Polle J.E.W., Lee H.K., Hyun S.M., Chang M. (2003). Xanthophylls in microalgae: From biosynthesis to biotechnological mass production and application. J. Microbiol. Biotechnol..

[67] Spolaore P., Joannis-Cassan C., Duran E., Isambert A. (2006). Commercial applications of microalgae. J. Biosci. Bioeng..

[68] Lee Y.-K. (1997). Commercial production of microalgae in the Asia-Pacific rim. J. Appl. Phycol..

[69] Borowitzka M.A. (1999). Commercial production of microalgae: Ponds, tanks, tubes and fermenters. J. Biotechnol..

[70] Carvalho A.P., Meireles L.A., Malcata F.X. (2006). Microalgal reactors: A review of enclosed system designs and performances. Biotechnol. Prog..

[71] Cornet J.F. (1998). Le technoscope: Les photobioréacteurs. Biofutur.

[72] Soletto D., Binaghi L., Lodi A., Carvalho J.C.M., Converti A. (2005). Batch and fed-batch cultivations of *Spirulina platensis* using ammonium sulphate and urea as nitrogen sources. Aquaculture.

[73] Yamaguchi K. (1996). Recent advances in microalgal bioscience in Japan, with special reference to utilization of biomass and metabolites: A review. J. Appl. Phycol..

[74] Liang S.Z., Liu X.M., Chen F., Chen Z.J. (2004). Current microalgal health food R & D activities in China. Hydrobiologia.

[75] Apt K.E., Behrens P.W. (1999). Commercial developments in microalgal biotechnology. J. Phycol..

[76] Iwamoto K., Shiraiwa Y. (2005). Salt-regulated mannitol metabolism in algae. Mar. Biotechnol..

[77] Metting F.B. (1996). Biodiversity and application of microalgae. J. Ind. Microbiol. Biotechnol..

[78] Del Campo J.A., Rodriguez H., Moreno J., Vargas M.A., Guerrero M.G. (2001). Lutein production by *Muriellopsis* sp. in an outdoor tubular photobioreactor. J. Biotechnol..

[79] Kobayashi M., Kakizono T., Yamaguchi K., Nishio N., Nagai S. (1992). Growth and astaxanthin formation of *Haematococcus pluvialis* in heterotrophic and mixotrophic conditions. J. Ferment. Bioeng..

[80] Boussiba S. (2000). Carotenogenesis in the green alga *Haematococcus pluvialis*: Cellular physiology and stress response. Physiol. Plant..

[81] Radmer R.J. (1996). Algal diversity and commercial algal products. BioScience.

[82] Rangel-Yagui C.d.O., Danesi E.D.G., de Carvalho J.C.M., Sato S. (2004). Chlorophyll production from *Spirulina platensis*: Cultivation with urea addition by fed-batch process. Bioresour. Technol..

[83] Jensen G.S., Ginsberg D.I., Drapeau C. (2001). Blue-green algae as an immuno-enhancer and biomodulator. J. Am. Nutraceut. Assoc..

[84] Benedetti S., Benvenuti F., Pagliarani S., Francogli S., Scoglio S., Canestrari F. (2004). Antioxidant properties of a novel phycocyanin extract from the blue-green alga *Aphanizomenon flos-aquae*. Life Sci..

[85] Borowitzka M.A. (1997). Microalgae for aquaculture: Opportunities and constraints. J. Appl. Phycol..

[86] Muller-Feuga A. (2000). The role of microalgae in aquaculture: Situation and trends. J. Appl. Phycol..

[87] Brown M.R., Mular M., Miller I., Farmer C., Trenerry C. (1999). The vitamin content of microalgae used in aquaculture. J. Appl. Phycol..

[88] Renaud S.M., Thinh L.V., Lambrinidis G., Parry D.L. (2002). Effect of temperature on growth, chemical composition and fatty acid composition of tropical Australian microalgae grown in batch cultures. Aquaculture.

[89] Stolz P., Obermayer B. (2005). Manufacturing microalgae for skin care. Cosmet. Toilet..

[90] Sekar S., Chandramohan M. (2008). Phycobiliproteins as a commodity: Trends in applied research, patents and commercialization. J. Appl. Phycol..

[91] Pushparaj B., Pelosi E., Carlozzi P., Torzillo G. (1995). Yield and biochemical-composition of a marine cyanobacterium (*Nodularia* sp.) in outdoor culture. Aquat. Microb. Ecol..

[92] Vonshak A., Chanawongse L., Bunnag B., Tanticharoen M. (1996). Light acclimation and photoinhibition in three *Spirulina platensis* (cyanobacteria) isolates. J. Appl. Phycol..

[93] Torzillo G., Bernardini P., Masojidek J. (1998). On-line monitoring of chlorophyll fluorescence to assess the extent of photoinhibition of photosynthesis induced by high oxygen concentration and low temperature and its effect on the productivity of outdoor cultures of *Spirulina platensis* (Cyanobacteria). J. Phycol..

[94] Molina E., Fernandez J., Acien F.G., Chisti Y. (2001). Tubular photobioreactor design for algal cultures. J. Biotechnol..

[95] Roy L.A., Davis D.A., Saoud I.P. (2006). Effects of lecithin and cholesterol supplementation to practical diets for *Litopenaeus vannamei* reared in low salinity waters. Aquaculture.

[96] Ugwu C.U., Aoyagi H., Uchiyama H. (2007). Influence of irradiance, dissolved oxygen concentration, and temperature on the growth of *Chlorella sorokiniana*. Photosynthetica.

[97] Masojídek J., Vonshak A., Torzillo G., Suggett D.J., Prášil O., Borowitzka M.A. (2010). Chlorophyll Fluorescence Applications in Microalgal Mass Cultures. Chlorophyll a Fluorescence in Aquatic Sciences: Methods and Applications.

[98] Giovagnetti V., Brunet C., Conversano F., Tramontano F., Obernosterer I., Ridame C., Guieu C. (2013). Assessing the role of dust deposition on phytoplankton ecophysiology and succession in a low-nutrient low-chlorophyll ecosystem: A mesocosm experiment in the Mediterranean Sea. BioGeosciences.

[99] Pulz O. (2001). Photobioreactors: Production systems for phototrophic microorganisms. Appl. Microbiol. Biotechnol..

[100] Xu L., Weathers P.J., Xiong X.-R., Liu C.-Z. (2009). Microalgal bioreactors: Challenges and opportunities. Eng. Life Sci..

[101] Litchman E., Klausmeier C.A. (2008). Trait-based Community Ecology of Phytoplankton. Ann. Rev. Ecol. Evol. Syst..

[102] Kooistra W.H.C.F., Gersonde R., Medlin L.K., Mann D.G., Paul G.F., Andrew H.K. (2007). The Origin and Evolution of the Diatoms: Their Adaptation to a Planktonic Existence. Evolution of Primary Producers in the Sea.

[103] Mann D.G., Droop S.J.M. (1996). Biodiversity, biogeography and conservation of diatoms. Hydrobiologia.

[104] Amato A., Kooistra W.H.C.F., Ghiron J.H.L., Mann D.G., Proschold T., Montresor M. (2007). Reproductive isolation among sympatric cryptic species in marine diatoms. Protist.

[105] Amato A., Montresor M. (2008). Morphology, phylogeny, and sexual cycle of *Pseudo-nitzschia mannii* sp. nov. (Bacillariophyceae): A pseudo-cryptic species within the *P. pseudodelicatissima* complex. Phycologia.

[106] Medlin L.K., Kooistra W.H.C.F. (2010). Methods to estimate the diversity in the marine photosynthetic protist community with illustrations from case studies: A review. Diversity.

[107] Falkowski P.G., Katz M.E., Knoll A.H., Quigg A., Raven J.A., Schofield O., Taylor F.J.R. (2004). The evolution of modern eukaryotic phytoplankton. Science.

[108] Bowler C., Allen A.E., Badger J.H., Grimwood J., Jabbari K., Kuo A., Maheswari U., Martens C., Maumus F., Otillar R.P. (2008). The *Phaeodactylum* genome reveals the evolutionary history of diatom genomes. Nature.

[109] Tirichine L., Bowler C. (2011). Decoding algal genomes: Tracing back the history of photosynthetic life on Earth. Plant. J..

[110] Reusch T.B.H., Boyd P.W. (2013). Experimental evolution meets marine phytoplankton. Evolution.

[111] Groben R., John U., Eller G., Lange M., Medlin L.K. (2004). Using fluorescently-labelled rRNA probes for hierarchical estimation of phytoplankton diversity—A mini-review. Nova Hedwig..

[112] Orsini L., Procaccini G., Sarno D., Montresor M. (2004). Multiple rDNA ITS-types within the diatom *Pseudo-nitzschia delicatissima* (Bacillariophyceae) and their relative abundances across a spring bloom in the Gulf of Naples. Mar. Ecol. Prog. Ser..

[113] Massana R., Castresana J., Balagué V., Guillou L., Romari K., Groisillier A., Valentin K., Pedrós-Alió C. (2004). Phylogenetic and ecological analysis of novel marine stramenopiles. Appl. Environ. Microbiol..

[114] Sarno D., Kooistra W.H.C.F., Medlin L.K., Percopo I., Zingone A. (2005). Diversity in the genus *Skeletonema* (bacillariophyceae). (ii) An assessment of the taxonomy of *S. costatum* like species with the description of four new species. J. Phycol..

[115] Smayda T.J. (1970). The suspension and sinking of phytoplankton in the sea. Oceanogr. Mar. Biol. Annu. Rev..

[116] Pickett-Heaps J.D. (1998). Cell division and moprhogenesis of the centric diatom *Chaetoceros decipiens* (bacillariophyceae) I. Living cells. J. Phycol..

[117] Yokota K., Sterner R.W. (2011). Trade-offs limiting the evolution of coloniality: Ecological displacement rates used to measure small costs. Proc. Biol. Sci..

[118] Rousseau V., Chrétiennot-Dinet M.-J., Jacobsen A., Verity P., Whipple S. (2007). The life cycle of Phaeocystis: State of knowledge and presumptive role in ecology. Biogeochemistry.

[119] Young A.M., Karp-Boss L., Jumars P.A., Landis E.N. (2012). Quantifying diatom aspirations: Mechanical properties of chain-forming species. Limnol. Oceanogr..

[120] Smetacek V., Assmy P., Henjes J. (2004). The role of grazing in structuring Southern Ocean pelagic ecosystems and biogeochemical cycles. Antarct. Sci..

[121] Bergkvist J., Thor P., Jakobsen H.H., Wangberg S.-A., Selander E. (2012). Grazer-induced chain length plasticity reduces grazing risk in a marine diatom. Limnol. Oceanogr..

[122] Karp-Boss L., Boss E., Jumars P.A. (1996). Nutrient fluxes to planktonic osmotrophs in the presence of fluid motion. Oceanogr. Mar. Biol. Annu. Rev..

[123] Finkel Z.V., Beardall J., Flynn K.J., Quigg A., Rees T.A.V., Raven J.A. (2010). Phytoplankton in a changing world: Cell size and elemental stoichiometry. J. Plankton Res..

[124] Not F., Massana R., Latasa M., Marie D., Colson C., Eikrem W., Pedros-Alio C., Vaulot D., Simon N. (2005). Late summer community composition and abundance of photosynthetic picoeukaryotes in Norwegian and Barents Seas. Limnol. Oceanogr..

[125] Worden A.Z., Not F., Kirchman D.L. (2008). Ecology and Diversity of Picoeukaryotes. Microbial Ecology of the Oceans.

[126] Raven J.A. (1998). The twelfth Tansley Lecture. Small is beautiful: The picophytoplankton. Funct. Ecol..

[127] Raven J.A., Finkel Z.V., Irwin A.J. (2005). Picophytoplankton: Bottom-up and top-down controls on ecology and evolution. Vie Milieu Life Environ..

[128] Palenik B., Grimwood J., Aerts A., Rouze P., Salamov A., Putnam N., Dupont C., Jorgensen R., Derelle E., Rombauts S. (2007). The tiny eukaryote *Ostreococcus* provides genomic insights into the paradox of plankton speciation. Proc. Natl. Acad. Sci. USA.

[129] Six C., Finkel Z.V., Rodriguez F., Marie D., Partensky F., Campbell D.A. (2008). Contrasting photoacclimation costs in ecotypes of the marine eukaryotic picoplankter *Ostreococcus*. Limnol. Oceanogr..

[130] Six C., Sherrard R., Lionard M., Roy S., Campbell D.A. (2009). Photosystem II and pigment dynamics among ecotypes of the green alga *Ostreococcus*. Plant Physiol..

[131] Cox E.R. (1980). Phytoflagellates.

[132] MacIntyre H., Geider R., Miller D. (1996). Microphytobenthos: The ecological role of the “secret garden” of unvegetated, shallow-water marine habitats. I. Distribution, abundance and primary production. Estuaries.

[133] Hallegraeff G.M. (1993). A review of harmful algal blooms and their apparent global increase. Phycologia.

[134] Chepurnov V.A., Mann D.G., Sabbe K., Vyverman W., Jeon K.W. (2004). Experimental Studies an Sexual Reproduction in Diatoms. International Review of Cytology—A Survey of Cell Biology.

[135] Berman-Frank I., Quigg A., Finkel Z.V., Irwin A.J., Haramaty L. (2007). Nitrogen-fixation strategies and Fe requirements in cyanobacteria. Limnol. Oceanogr..

[136] Flynn K.J., Stoecker D.K., Mitra A., Raven J.A., Glibert P.M., Hansen P.J., Granéli E., Burkholder J.M. (2013). Misuse of the phytoplankton–zooplankton dichotomy: The need to assign organisms as mixotrophs within plankton functional types. J. Plankton Res..

[137] Wilken S., Huisman J., Naus-Wiezer S., Donk E. (2013). Mixotrophic organisms become more heterotrophic with rising temperature. Ecol. Lett..

[138] Stoecker D.K. (1998). Conceptual models of mixotrophy in planktonic protists and some ecological and evolutionary implications. Eur. J. Protistol..

[139] Falkowski P.G. (1983). Light-shade adaptation and vertical mixing of marine-phytoplankton—A comparative field-study. J. Mar. Res..

[140] Marchetti A., Maldonado M.T., Lane E.S., Harrison P.J. (2006). Iron requirements of the pennate diatom *Pseudo*-*nitzschia*: Comparison of oceanic (high-nitrate, low-chlorophyll waters) and coastal species. Limnol. Oceanogr..

[141] Hare C.E., Leblanc K., DiTullio G.R., Kudela R.M., Zhang Y., Lee P.A., Riseman S., Hutchins D.A. (2007). Consequences of increased temperature and CO_2_ for phytoplankton community structure in the Bering Sea. Mar. Ecol. Prog. Ser..

[142] Toseland A., Daines S.J., Clark J.R., Kirkham A., Strauss J., Uhlig C., Lenton T.M., Valentin K., Pearson G.A., Moulton V. (2013). The impact of temperature on marine phytoplankton resource allocation and metabolism. Nat. Clim. Chang..

[143] Takano H., Furuune H., Burgess J.G., Manabe E., Hirano M., Okazaki M., Matsunaga T. (1993). Production of ultrafine calcite particles by coccolithophorid algae grown in a biosolar reactor supplied with sunlight. Appl. Biochem. Biotechnol..

[144] DasSarma P., Coker J.A., Huse V., DasSarma S. (2010). Halophiles, Industrial Applications. Encyclopedia of Industrial Biotechnology.

[145] Berges J.A., Franklin D.J., Harrison P.J. (2001). Evolution of an artificial seawater medium: Improvements in enriched seawater, artificial water over the last two decades. J. Phycol..

[146] Keller M.D., Selvin R.C., Claus W., Guillard R.R.L. (1987). Media for the culture of oceanic ultraphytoplankton. J. Phycol..

[147] The National Center for Marine Algae and Microbiota (NCMA) Culturing Diversity. https://ncma.bigelow.org.

[148] Guillard R.R., Ryther J.H. (1962). Studies of marine planktonic diatoms. I. *Cyclotella nana* Hustedt, and *Detonula confervacea* (cleve) Gran. Can. J. Microbiol..

[149] Guillard R.R.L., Smith W.L., Chanley M.H. (1975). Culture of Phytoplankton for Feeding Marine Invertebrates. Culture of Marine Invertebrate Animals.

[150] Keller M.D., Guillard R.R.L., Anderson D.M., White A.W., Baden D.G. (1985). Factors Significant to Marine Diatom Culture. Toxic Dinoflagellates.

[151] Moore L.R., Coe A., Zinser E.R., Saito M.A., Sullivan M.B., Lindell D., Frois-Moniz K., Waterbury J., Chisholm S.W. (2007). Culturing the marine cyanobacterium Prochlorococcus. Limnol. Oceanogr. Methods.

[152] Noel M.H., Kawachi M., Inouye I. (2004). Induced dimorphic life cycle of a coccolithophorid, *Calyptrosphaera sphaeroidea* (Prymnesiophyceae, Haptophyta). J. Phycol..

[153] Andersen T., Schartau A., Paasche E. (1991). Quantifying external and internal nitrogen and phosphorus pools, as well as nitrogen and phosphorus supplied through remineralization, in coastal marine plankton by means of a dilution technique. Mar. Ecol. Prog. Ser..

[154] Irwin A.J., Finkel Z.V., Schofield O.M.E., Falkowski P.G. (2006). Scaling-up from nutrient physiology to the size-structure of phytoplankton communities. J. Plankton Res..

[155] Verdy A., Follows M., Flierl G. (2009). Optimal phytoplankton cell size in an allometric model. Mar. Ecol. Prog. Ser..

[156] Shuter B.J. (1978). Size dependence of phosphorus and nitrogen subsistence quotas in unicellular microorganisms. Limnol. Oceanogr..

[157] Sommer U. (1984). The paradox of the plankton: Fluctuations of phosphorus availability maintain diversity of phytoplankton in flow-through cultures. Limnol. Oceanogr..

[158] Morel F.M.M. (1987). Kinetics of nutrient-uptake and growth in phytoplankton. J. Phycol..

[159] Smayda T.J. (1997). Harmful algal blooms: Their ecophysiology and general relevance to phytoplankton blooms in the sea. Limnol. Oceanogr..

[160] Litchman E., Klausmeier C.A., Bossard P. (2004). Phytoplankton nutrient competition under dynamic light regimes. Limnol. Oceanogr..

[161] Litchman E., Klausmeier C.A., Schofield O.M., Falkowski P.G. (2007). The role of functional traits and trade-offs in structuring phytoplankton communities: Scaling from cellular to ecosystem level. Ecol. Lett..

[162] Eppley R.W., Carlucci A.F., Holm-Hansen O., Kiefer D., McCarthy J.J., Venrick E., Williams P.M. (1971). Phytoplankton growth and composition in shipboard cultures supplied with nitrate, ammonium, or urea as nitrogen source. Limnol. Oceanogr..

[163] Karthikeyan P., Manimaran K., Sampathkumar P., Rameshkumar L. (2013). Growth and nutrient removal properties of the diatoms, *Chaetoceros curvisetus* and *C. simplex* under different nitrogen sources. Appl. Water Sci..

[164] Chandrasekaran R., Barra L., Caruso T., Corsaro L., Piaz F.D., Graziani G., Corato F., Brunet C. (2014). Light modulation on the biochemical properties of the diatom *Skeletonema marinoi*: Relevance of photosynthetic manipulation in the biotechnological field.

[165] Allen A.E., Dupont C.L., Obornik M., Horak A., Nunes-Nesi A., McCrow J.P., Zheng H., Johnson D.A., Hu H., Fernie A.R. (2011). Evolution and metabolic significance of the urea cycle in photosynthetic diatoms. Nature.

[166] Joy K.W., Hageman R.H. (1966). The purification and properties of nitrite reductase from higher plants, and its dependence on ferredoxin. Biochem. J..

[167] Flynn K.J., Fasham M.J.R., Hipkin C.R. (1997). Modelling the interactions between ammonium and nitrate uptake in marine phytoplankton. Phil. Trans. R Soc. B Biol. Sci..

[168] Thomas W.H., Hastings J., Fujita M. (1980). Ammonium input to the sea via large sewage outfalls-Part 2: Effects of ammonium on growth and photosynthesis of southern California phytoplankton cultures. Mar. Environ. Res..

[169] Eppley R.W., Rogers J.N., McCarthy J.J. (1969). Half-saturation constants for uptake of nitrate and ammonium by marine-phytoplankton. Limnol. Oceanogr..

[170] Kamp A., de Beer D., Nitsch J.L., Lavik G., Stief P. (2011). Diatoms respire nitrate to survive dark and anoxic conditions. Proc. Natl. Acad. Sci. USA.

[171] Kamp A., Stief P., Knappe J., de Beer D. (2013). Response of the ubiquitous pelagic diatom *Thalassiosira weissflogii* to darkness and anoxia. PLoS One.

[172] Alonso D.L., Belarbi E.-H., Fernández-Sevilla J.M., Rodríguez-Ruiz J., Grima E.M. (2000). Acyl lipid composition variation related to culture age and nitrogen concentration in continuous culture of the microalga *Phaeodactylum tricornutum*. Phytochemistry.

[173] Reitan K.I., Rainuzzo J.R., Olsen Y. (1994). Effect of nutrient limitation on fatty acid and lipid content of marine microalgae. J. Phycol..

[174] Lombardi A.T., Wangersky P.J. (1991). Influence of phosphorus and silicon on lipia class production by the marine diatom *Chaetoceros gracilis* grown in turbidostat cage cultures. Mar. Ecol. Prog. Ser..

[175] Boussiba S., Vonshak A. (1991). Astaxanthin accumulation in the green-alga *Haematococcus pluvialis*. Plant Cell Physiol..

[176] Ben-Amotz A., Avron M. (1983). On the factors which determine the massive bcarotene accumulation in the halotolerant alga *Dunaliella bardawil*. Plant Physiol..

[177] Falkowski P.G., Sukenik A., Herzig R. (1989). Nitrogen limitation in *Isochrysis galbana* (haptophyceae). ii. Relative abundance of chloroplast proteins. J. Phycol..

[178] Herrig R., Falkowski P.G. (1989). Nirogen limitation in *Isochrysis galbana* (haptophyceae). I. Photosynthetic energy conversion and growth efficiencies. J. Phycol..

[179] Geider R.J., La Roche J., Greene R.M., Olaizola M. (1993). Response of the photosynthetic apparatus of *Phaeodactylum tricornutum* (bacillariophyceae) to nitrate, phosphate, or iron starvation. J. Phycol..

[180] Geider R.J., Macintyre H.L., Graziano L.M., McKay R.M.L. (1998). Responses of the photosynthetic apparatus of *Dunaliella tertiolecta* (Chlorophyceae) to nitrogen and phosphorus limitation. Eur. J. Phycol..

[181] Lai J., Yu Z., Song X., Cao X., Han X. (2011). Responses of the growth and biochemical composition of *Prorocentrum donghaiense* to different nitrogen and phosphorus concentrations. J. Exp. Mar. Biol. Ecol..

[182] Liu Y., Song X., Cao X., Yu Z. (2013). Responses of photosynthetic characters of *Skeletonema costatum* to different nutrient conditions. J. Plankton Res..

[183] Beardall J., Johnston A., Raven J. (1998). Environmental regulation of CO_2_ concentrating mechanisms in microalgae. Can. J. Bot..

[184] Martin J.H. (1991). Iron, Liebig’s Law, and the greenhouse. Oceanography.

[185] Dixon J.L. (2008). Macro and micro nutrient limitation of microbial productivity in oligotrophic subtropical Atlantic waters. Environ. Chem..

[186] Strzepek R.F., Harrison P.J. (2004). Photosynthetic architecture differs in coastal and oceanic diatoms. Nature.

[187] Raven J.A. (1988). The iron and molybdenum use efficiencies of plant-growth with different energy, carbon and nitrogen-sources. New Phytol..

[188] Maldonado M.T., Price N.M. (1996). Influence of N substrate on Fe requirements of marine centric diatoms. Mar. Ecol. Prog. Ser..

[189] Sunda W., Huntsman S.A. (1997). Interrelated influence of iron, light and cell size on marine phytoplankton growth. Nature.

[190] Morel F.M.M., Reinfelder J.R., Roberts S.B., Chamberlain C.P., Lee J.G., Yee D. (1994). Zinc and carbon co-limitation of marine-phytoplankton. Nature.

[191] Raven J.A., Evans M.C.W., Korb R.E. (1999). The role of trace metals in photosynthetic electron transport in O_2_ evolving organisms. Photosynth. Res..

[192] Price N.M., Morel F.M.M. (1991). Co-limitation of phytoplankton growth by nickel and nitrogen. Limnol. Oceanogr..

[193] Provasoli L., Carlucci A.F., Stewart W.D.P., Abbott M.R (1974). Vitamins and Growth Regulators. Algal Physiology and Biochemistry.

[194] Adolf J.E., Stoecker D.K., Harding L.W. (2006). The balance of autotrophy and heterotrophy during mixotrophic growth of *Karlodinium micrum* (Dinophyceae). J. Plankton Res..

[195] Hammer A.C., Pitchford J.W. (2005). The role of mixotrophy in plankton bloom dynamics, and the consequences for productivity. ICES J. Mar. Sci..

[196] Ward B.A., Dutkiewicz S., Barton A.D., Follows M.J. (2011). Biophysical aspects of resource acquisition and competition in algal mixotrophs. Am. Nat..

[197] Ren H.-Y., Liu B.-F., Ma C., Zhao L., Ren N.-Q. (2013). A new lipid-rich microalga Scenedesmus sp. strain R-16 isolated using Nile red staining: Effects of carbon and nitrogen sources and initial pH on the biomass and lipid production. Biotechnol. Biofuels.

[198] Ketchum B.H. (1939). The development and restoration of deficiencies in the phosphorus and nitrogen composition of unicellular plants. J. Cell. Comp. Physiol..

[199] Syrett P.J., Lewin R.A. (1962). Nitrogen Assimilation. Physiology and Biochemistry of Algae.

[200] Terry K.L., Hirata J., Laws E.A. (1983). Light-limited growth of two strains of the marine diatom *Phaeodactylum tricornutum* Bohlin: Chemical composition, carbon partitioning and the diel peridiocity of physiological processes. J. Exp. Mar. Biol. Ecol..

[201] Curtis P.J., Megard R.O. (1987). Interactions among irradiance, oxygen evolution and nitrite uptake by *Chlamydomonas* (chlorophyceae). J. Phycol..

[202] Kerby N.W., Rowell P., Stewart W.D.P., Cresswell R.C., Rees T.A.V., Shah N. (1989). The Transport, Assimilation and Production of Nitrogenous Compounds by Cyanobacteria and Microalgae. Algal and Cyanobacterial Biotechnology.

[203] Meseck S.L., Alix J.H., Wikfors G.H. (2005). Photoperiod and light intensity effects on growth and utilization of nutrients by the aquaculture feed microalga, *Tetraselmis chui* (PLY429). Aquaculture.

[204] Fabregas J., Maseda A., Dominguez A., Otero A. (2004). The cell composition of *Nannochloropsis* sp changes under different irradiances in semicontinuous culture. World J. Microbiol. Biotechnol..

[205] Klyachko-Gurvich G.L., Tsoglin L.N., Doucha J., Kopetskii J., Shebalina I.B., Semenenko V.E. (1999). Desaturation of fatty acids as an adaptive response to shifts in light intensity. Physiol. Plant..

[206] Long S.P., Humphries S., Falkowski P.G. (1994). Photoinhibition of photosynthesis in nature. Annu. Rev. Plant Physiol. Plant Biol..

[207] Franklin L.A., Osmond C.B., Larkum A.W.D., Larkum A.W.D., Douglas S.E., Raven J.A. (2003). Photoinhibition, UB-B and Algal Photosynthesis. Photosynthesis in Algae.

[208] Vonshak A., Torzillo G., Richmond A. (2004). Environmental Stress Physiology. Handbook of Microalgal Culture.

[209] Ralph P.J., Gademann R. (2005). Rapid light curves: A powerful tool to assess photosynthetic activity. Aquat. Bot..

[210] Lepetit B., Volke D., Gilbert M., Wilhelm C., Goss R. (2010). Evidence for the existence of one antenna-associated, lipid-dissolved and two protein-bound pools of diadinoxanthin cycle pigments in diatoms. Plant Physiol..

[211] Fábregas J., Dominguez A., Maseda A., Otero A. (2003). Interactions between irradiance and nutrient availability during astaxanthin accumulation and degradation in *Haematococcus pluvialis*. Appl. Microbiol. Biotechnol..

[212] García-Malea M.C., Acién F.G., Del Río E., Fernández J.M., Cerón M.C., Guerrero M.G., Molina-Grima E. (2009). Production of astaxanthin by *Haematococcus pluvialis*: Taking the one-step system outdoors. Biotechnol. Bioeng..

[213] Raven J.A., Geider R.J. (1988). Temperature and algal growth. New Phytol..

[214] Davison I.R. (1991). Environmental-effects on algal photosynthesis—Temperature. J. Phycol..

[215] Fogg G.E., Rai L.C., Gaur J.P. (2001). Algal Adaptation to Stress–Some General Remarks. Algal Adaptation to Environmental Stresses: Physiological, Biochemical and Molecular Mechanisms.

[216] Eppley R.W. (1972). Temperature and phytoplankton growth in the sea. Fish. Bull..

[217] Goldman J.C., Carpenter E.J. (1974). A kinetic approach to the effect of temperature on algal growth. Limnol. Oceanogr..

[218] Thompson P.A., Guo M., Harrison P.J. (1992). Effects of variation in temperature. I. On the biochemical composition of eight species of marine phytoplankton. J. Phycol..

[219] Thompson P.A., Guo M., Harrison P.J., Whyte J.N.C. (1992). Effects of variation in temperature.II. On the fatty acid composition of eight species of marine phytoplankton. J. Phycol..

[220] Gao Y., Smith G.J., Alberte R.S. (2000). Temperature dependence of nitrate reductase activity in marine phytoplankton: Biochemical analysis and ecological, implications. J. Phycol..

[221] Lomas M.W., Glibert P.M. (1999). Interactions between NH_4_^+^ and NO_3_^−^ uptake and assimilation: Comparison of diatoms and dinoflagellates at several growth temperatures. Mar. Biol..

[222] Lomas M.W., Glibert P.M. (1999). Temperature regulation of nitrate uptake: A novel hypothesis about nitrate uptake and reduction in cool-water diatoms. Limnol. Oceanogr..

[223] Payer H.D., Chiemvichak Y., Hosakul K., Kongpanichkul C., Kraidej L., Nguitragul M., Reungmanipytoon S., Buri P., Shelef G., Soeder C.J. (1980). Temperature as an important climatic factor during mass production of microscopic algae. Algae Biomass.

[224] De Oliveira M., Monteiro M., Robbs P., Leite S. (1999). Growth and chemical composition of *Spirulina maxima* and *Spirulina platensis* biomass at different temperatures. Aquac. Int..

[225] Marre E., Lewin R.A. (1962). Temperature. Physiology and Biochemistry of Algae.

[226] Teoh M.L., Chu W.L., Marchant H., Phang S.M. (2004). Influence of culture temperature on the growth, biochemical composition and fatty acid profiles of six Antarctic microalgae. J. Appl. Phycol..

[227] Nelson J.R., Guarda S., Cowell L.E., Heffernan P.B. (1992). Evaluation of microalgal clones for mass culture in a subtropical greenhouse bivalve hatchery: Growth-rates and biochemical composition at 30 °C. Aquaculture.

[228] Barsanti L., Gualtieri P. (2006). Algae: Anatomy, Biochemistry and Biotechnology.

[229] Thomas W.H., Tornabene T.G., Weissman J.C. (1984). Screening for Lipid Yielding Microalgae: Activities for 1983.

[230] Kirst G.O. (1977). Coordination of ionic relations and mannitol concentrations in the euryhaline unicellular alga, *Platymonas subcordiformis* (Hazen) after osmotic shocks. Planta.

[231] Strizh I., Popova L., Balnokin Y.V. (2004). Physiological aspects of adaptation of the marine microalga *Tetraselmis (Platymonas) viridis* to various medium salinity. Russ. J. Plant Physiol..

[232] Borowitzka L.J., Brown A.D. (1974). The salt relations of marine and halophilic species of the unicellular green alga, *Dunaliella*. The role of glycerol as a compatible solute. Arch. Mikrobiol..

[233] Kirst G.O., Lobban C.S., Chapman D.J., Kremer B.P. (1988). Turgor Pressure Regulation in Marine Macroalgae. Experimental Phycology.

[234] Reed R.H., Stewart W.D.P., Rogers L.J., Gallon J.R. (1988). The Responses of Cyanobacteria to Salt Stress. Biochemistry of the Algae and Cyanobacteria.

[235] Kirst G.O. (1990). Salinity tolerance of eukaryotic marine-algae. Annu. Rev. Plant Physiol. Plant Mol. Biol..

[236] Ben-Amotz A., Tornabene T.G., Thomas W.H. (1985). Chemical profile of selected species of microalgae with emphasis on lipids. J. Phycol..

[237] Renaud S.M., Parry D.L. (1994). Microalgae for use in tropical aquaculture II: Effect of salinity on growth, gross chemical composition and fatty acid composition of three species of marine microalgae. J. Appl. Phycol..

[238] Ahmad I., Hellebust J.A. (1993). Protein-biosynthesis in salt-shocked cells of *Stichococcus bacillaris* (chlorophyceae). J. Phycol..

[239] Chakraborty P., Acharyya T., Babu P.V.R., Bandyopadhyay D. (2011). Impact of salinity and pH on phytoplankton communities in a tropical freshwater system: An investigation with pigment analysis by HPLC. J. Environ. Monit..

[240] Liska A.J., Shevchenko A., Pick U., Katz A. (2004). Enhanced photosynthesis and redox energy production contribute to salinity tolerance in *Dunaliella* as revealed by homology-based proteomics. Plant Physiol..

[241] Borowitzka M.A., Borowitzka L.J., Kessly D. (1990). Effects of salinity increase on carotenoid accumulation in the green alga *Dunaliella salina*. J. Appl. Phycol..

[242] Ramos A., Polle J., Tran D., Cushman J.C., Jin E., Valera J. (2011). The unicellular green alga *Dunaliella salina* Teod. as a model for abiotic stress tolerance: Genetic advances and future perspectives. Algae.

[243] Emerson R., Green L. (1938). Effect of hydrogen-ion concentration on *Chlorella* photosynthesis. Plant Physiol..

[244] Moss B. (1973). The influence of environmental factors on the distribution of freshwater algae: An experimental study. II. The role of pH and the carbon dioxide- bicarbonate system. J. Ecol..

[245] Azov Y. (1982). Effect of pH on inorganic carbon uptake in algal cultures. Appl. Environ. Microbiol..

[246] Sunda W., Price N.M., Morel F.M.M., Andersen R.A. (2005). Trace Metal Ion Buffers and Their Use in Culture Studies. Algal Culturing Techniques.

[247] Meseck S.L., Smith B. (2004). How high pH’s can affect the chemistry in large volume cultures of *Tetraselmis chui* (PLY429). J. Shellfish Res..

[248] Smith B., Meseck S. (2004). Some implications of controlling CO_2_ supply to cultures of *Tetraselmis chui* (PLY429). J. Shellfish Res..

[249] Goldman J.C., Azov Y., Riley C.B., Dennett M.R. (1982). The effect of pH in intensive microalgal cultures.I. Biomass regulation. J. Exp. Mar. Biol. Ecol..

[250] Goldman J.C., Riley C.B., Dennett M.R. (1982). The effect of pH in intensive microalgal cultures. II. Species competition. J. Exp. Mar. Biol. Ecol..

[251] Dimier C., Brunet C., Geider R., Raven J. (2009). Growth and photoregulation dynamics of the picoeukaryote *Pelagomonas calceolata* in fluctuating light. Limnol. Oceanogr..

[252] Schellenberger Costa B., Sachse M., Jungandreas A., Bartulos C.R., Gruber A., Jakob T., Kroth P.G., Wilhelm C. (2013). Aureochrome 1a is involved in the photoacclimation of the diatom *Phaeodactylum tricornutum*. PLoS One.

[253] Schellenberger Costa B., Jungandreas A., Jakob T., Weisheit W., Mittag M., Wilhelm C. (2013). Blue light is essential for high light acclimation and photoprotection in the diatom *Phaeodactylum tricornutum*. J. Exp. Bot..

[254] Torkamani S., Wani S.N., Tang Y.J., Sureshkumar R. (2010). Plasmon-enhanced microalgal growth in miniphotobioreactors. Appl. Phys. Lett..

[255] Perrine Z., Negi S., Sayre R.T. (2012). Optimization of photosynthetic light energy utilization by microalgae. Algal Res..

[256] Murchie E.H., Pinto M., Horton P. (2009). Agriculture and the new challenges for photosynthesis research. New Phytol..

[257] Simionato D., Sforza E., Carpinelli E.C., Bertucco A., Giacometti G.M., Morosinotto T. (2011). Acclimation of Nannochloropsis gaditana to different illumination regimes: Effects on lipids accumulation. Bioresour. Technol..

[258] Van Leeuwe M.A., van Sikkelerus B., Gieskes W.W.C., Stefels J. (2005). Taxon-specific differences in photoacclimation to fluctuating irradiance in an Antarctic diatom and a green flagellate. Mar. Ecol. Prog. Ser..

[259] Litchman E. (2000). Growth rates of phytoplankton under fluctuating light. Freshw. Biol..

[260] Grobbelaar J.U. (1989). Do light/dark cycles of medium frequency enhance phytoplankton productivity?. J. Appl. Phycol..

[261] Sforza E., Simionato D., Giacometti G.M., Bertucco A., Morosinotto T. (2012). Adjusted light and dark cycles can optimize photosynthetic efficiency in algae growing in photobioreactors. PLoS One.

[262] Paasche E. (1968). Marine plankton algae grown with light-dark cycles. II. *Ditylum brightwellii* and *Nitzschia turgidula*. Physiol. Plant..

[263] Brunet C., Davoult D., Casotti R. (1996). Physiological reactions to a change in light regime in cultured *Skeletonema costatum* (Bacillariophyta): Implications for estimation of phytoplankton biomass. Hydrobiologia.

[264] Jacquet S., Partensky F., Lennon J.F., Vaulot D. (2001). Diel patterns of growth and division in phytoplankton in culture. J. Phycol..

[265] Clark D.R., Flynn K.J., Owens N.J.P. (2002). The large capacity for dark nitrate-assimilation in diatoms may overcome nitrate limitation of growth. New Phytol..

[266] Ashworth J., Coesel S., Lee A., Armbrust E.V., Orellana M.V., Baliga N.S. (2013). Genome-wide diel growth state transitions in the diatom *Thalassiosira pseudonana*. Proc. Natl. Acad. Sci. USA.

[267] Wagner H., Jakob T., Wilhelm C. (2006). Balancing the energy flow from captured light to biomass under fluctuating light conditions. New Phytol..

[268] Sanchez-Saavedra M.P., Voltolina D. (2006). The growth rate, biomass production and composition of *Chaetoceros* sp. grown with different light sources. Aquac. Eng..

[269] Marchetti A., Schruth D.M., Durkin C.A., Parker M.S., Kodner R.B., Berthiaume C.T., Morales R., Allen A.E., Armbrust E.V. (2012). Comparative metatranscriptomics identifies molecular bases for the physiological responses of phytoplankton to varying iron availability. Proc. Natl. Acad. Sci. USA.

[270] Huysman M.J.J., Fortunato A.E., Matthijs M., Costa B.S., Vanderhaeghen R., van den Daele H., Sachse M., Inze D., Bowler C., Kroth P.G. (2013). AUREOCHROME1a-mediated induction of the diatom-specific cyclin dsCYC2 controls the onset of cell division in diatoms (*Phaeodactylum tricornutum*). Plant Cell.

[271] Shihira-Ishikawa I., Nakamura T., Higashi S.-i., Watanabe M. (2007). Distinct responses of chloroplasts to blue and green laser microbeam irradiations in the centric diatom *Pleurosira laevis*. Photochem. Photobiol..

[272] Cao S., Wang J., Chen D. (2013). Settlement and cell division of diatom *Navicula* can be influenced by light of various qualities and intensities. J. Basic Microbiol..

[273] Brunet C., Chandrasekaran R., Barra L., Giovagnetti V., Corato F., Ruban A.V. (2014). Spectral radiation dependent photoprotective mechanism in the diatom *Pseudo-nitzschia multistriata*. PLoS One.

[274] Brunet C., Conversano F., Margiotta F., Dimier C., Polimene L., Tramontano F., Saggiomo V. (2013). Role of light and photophysiological properties on phytoplankton succession during the spring bloom in the north-western Mediterranean Sea. Adv. Oceanogr. Limnol..

[275] Casotti R., Mazza S., Brunet C., Vantrepotte V., Ianora A., Miralto A. (2005). Growth inhibition and toxicity of the diatom aldehyde 2-trans, 4-trans-decadienal on *Thalassiosira weissflogii* (bacillariophyceae). J. Phycol..

[276] Wondraczek L., Batentschuk M., Schmidt M.A., Borchardt R., Scheiner S., Seemann B., Schweizer P., Brabec C.J. (2013). Solar spectral conversion for improving the photosynthetic activity in algae reactors. Nat. Commun..

[277] BioProject The National Center for Biotechnology Information (NCBI). http://www.ncbi.nlm.nih.gov/entrez/query.fcgi?DB=genomeprj.

[278] Organelle Genome Resources The National Center for Biotechnology Information (NCBI). http://www.ncbi.nlm.nih.gov/genomes/ORGANELLES/organelles.html.

[279] The Organelle Genome database (GOABASE). http://www.bch.umontreal.ca/gobase/gobase.html.

[280] Palenik B., Brahamsha B., Larimer F.W., Land M., Hauser L., Chain P., Lamerdin J., Regala W., Allen E.E., McCarren J. (2003). The genome of a motile marine *Synechococcus*. Nature.

[281] Worden A.Z., Lee J.-H., Mock T., Rouzé P., Simmons M.P., Aerts A.L., Allen A.E., Cuvelier M.L., Derelle E., Everett M.V. (2009). Green evolution and dynamic adaptations revealed by genomes of the marine picoeukaryotes Micromonas. Science.

[282] Vaulot D., Le Gall F., Marie D., Guillou L., Partensky F. (2004). The Roscoff Culture Collection (RCC): A collection dedicated to marine picoplankton. Nova Hedwig..

[283] Roscoff Culture Collection (RCC, Biological Station, Roscoff, France). http://www.sb-roscoff.fr/Phyto/RCC/index.php?option=com_frontpage&Itemid=1.

[284] Marine Microbial Eukaryote Transcriptome Sequencing Project. http://marinemicroeukaryotes.org/project_organisms.

[285] Algae Industry Magazine. http://www.algaeindustrymagazine.com.

[286] Dunahay T.G., Jarvis E.E., Roessler P.G. (1995). Genetic transformation of the diatoms *Cyclotella cryptica* and *Navicula saprophila*. J. Phycol..

[287] Falciatore A., Casotti R., Leblanc C., Abrescia C., Bowler C. (1999). Transformation of nonselectable reporter genes in marine diatoms. Mar. Biotechnol..

[288] Jin E., Polle J.E., Melis A. (2001). Involvement of zeaxanthin and of the Cbr protein in the repair of photosystem II from photoinhibition in the green alga *Dunaliella salina*. BBA Bioenerg..

[289] Zaslavskaia L., Lippmeier J., Shih C., Ehrhardt D., Grossman A., Apt K. (2001). Trophic conversion of an obligate photoautotrophic organism through metabolic engineering. Science.

[290] Doetsch N.A., Favreau M.R., Kuscuoglu N., Thompson M.D., Hallick R.B. (2001). Chloroplast transformation in *Euglena gracilis*: Splicing of a group III twintron transcribed from a transgenic psbK operon. Curr. Genet..

[291] Koksharova O., Wolk C. (2002). Genetic tools for cyanobacteria. Appl. Microbiol. Biotechnol..

[292] Nogales J., Gudmundsson S., Thiele I. (2013). Toward systems metabolic engineering in cyanobacteria: Opportunities and bottlenecks. Bioengineered.

[293] Sayre R.T., Wagner R.E., Siripornadulsil S., Farias C. (2012). Transgenic Algae for Delivering Antigens to an Animal. Patent.

[294] Geng D.G., Wang Y.Q., Wang P., Li W.B., Sun Y.R. (2003). Stable expression of hepatitis B surface antigen gene in *Dunaliella salina* (Chlorophyta). J. Appl. Phycol..

[295] Sun M., Qian K.X., Su N., Chang H.Y., Liu J.X., Chen G.F. (2003). Foot-and-mouth disease virus VP1 protein fused with cholera toxin B subunit expressed in *Chlamydomonas reinhardtii* chloroplast. Biotechnol. Lett..

[296] Mayfield S.P., Franklin S.E., Lerner R.A. (2003). Expression and assembly of a fully active antibody in algae. Proc. Natl. Acad. Sci. USA.

[297] Melis A., Happe T. (2001). Hydrogen production. Green algae as a source of energy. Plant Physiol..

[298] Rasala B.A., Mayfield S.P. (2011). The microalga *Chlamydomonas reinhardtii* as a platform for the production of human protein therapeutics. Bioengineered.

[299] Chen Y., Wang Y., Sun Y., Zhang L., Li W. (2001). Highly efficient expression of rabbit neutrophil peptide-1 gene in Chlorella ellipsoidea cells. Curr. Genet..

[300] Hempel F., Lau J., Klingl A., Maier U.G. (2011). Algae as protein factories: Expression of a human antibody and the respective antigen in the diatom *Phaeodactylum tricornutum*. PLoS One.

[301] Jinkerson R.E., Radakovits R., Posewitz M.C. (2013). Genomic insights from the oleaginous model alga Nannochloropsis gaditana. Bioengineered.

[302] Pan K., Qin J., Li S., Dai W., Zhu B., Jin Y., Yu W., Yang G., Li D. (2011). Nuclear monoploidy and asexual propagation of *Nannochloropsis oceanica* (eustigmatophyceae) as revealed by its genome sequence. J. Phycol..

[303] Depauw F.A., Rogato A., d’Alcalá M.R., Falciatore A. (2012). Exploring the molecular basis of responses to light in marine diatoms. J. Exp. Bot..

